# Pathways to the diagnosis of lung cancer in the UK: a cohort study

**DOI:** 10.1186/1471-2296-9-31

**Published:** 2008-05-18

**Authors:** Jacqueline Barrett, William Hamilton

**Affiliations:** 1 CAPER research unit, Exeter EX4 5BW, UK; 2 Academic Unit of Primary health Care, University of Bristol, 25-27 Belgrave Road, Bristol, BS8 2AA, UK

## Abstract

**Background:**

Lung cancer is the commonest cause of cancer death in the UK. Patients generally present to their general practitioner, but the pathway of diagnosis from first symptom to diagnosis has not been mapped. We performed a cohort study of 246 patients with lung cancer in Exeter, Devon UK. All patients had their cancer symptoms, referrals and diagnoses identified and dated using their doctors' records.

**Results:**

Three main routes to diagnosis emerged. The first was the expected route of outpatient referral; 150 (61% of the cohort) of patients took this route, although only 110 (45% of the whole cohort, 73% of those referred to outpatients) were referred to a respiratory department. 56 (23%) were admitted as an emergency, having previously described a lung cancer symptom to their doctor. 26 patients (11%) had no symptom of lung cancer reported before their diagnosis. The interval from first symptom to referral was similar across the different pathways. However, the referral to diagnosis interval was longer in patients misdirected to other outpatient departments (66 days, interquartile range 37,110) than those sent to respiratory clinics (29 days, 17,61) or admitted as an emergency (16 days 8,40); p < 0.001.

**Conclusion:**

Only a minority of lung cancer patients follow the traditional route to diagnosis. Clinical and research efforts need to consider the alternative routes if they are to maximise their impact on speed of diagnosis.

## Background

Over 37,000 new lung cancers are diagnosed each year in the UK [1]. Mortality is very high, with lung cancer the leading cause of cancer deaths in the UK [1]. The poor survival reflects the intrinsically aggressive nature of the tumour, with the shortest doubling time of the common cancers, plus the fact that symptoms occur relatively late in the growth of the cancer [2]. Many patients also delay presenting their symptoms to their doctor, and the duration of symptoms is now recognised to be longer than previously thought [3,4]. Thus few patients are diagnosed at a stage when they could be offered curative surgery [5]. Furthermore, no screening test has been found to be effective, and none is near to implementation, though trials are in progress using spiral CT [5]. Most lung cancers present with symptoms, and in the UK, most of these patients present initially to their general practitioner (GP) [6].

Unlike for most other common cancers, there exists a primary care investigation for possible lung cancer which has reasonable performance characteristics, namely the chest X-ray, although false-negatives can occur in up to a quarter of primary care patients [7]. Theoretically, the availability of chest X-rays should increase the proportion of patients diagnosed by the conventional route of GP outpatient referral to a respiratory physician, who then makes a tissue diagnosis. This is utilised in the recommendations of the National Institute of Clinical Effectiveness (NICE), which suggest an X-ray for several clinical scenarios, such as haemoptysis or hoarseness [8]. However, it is possible that these guidelines only help to identify more clinically obvious lung cancers. The level of compliance with guidance is unknown, and no reduction in lung cancer mortality has been seen following its introduction [9]. Given the absence of a screening test, and the uncomfortable fact that current guidance seems largely to identify those with late staging, research efforts are being targeted at the early symptoms of cancer [10]. Precise targeting of such efforts requires an understanding of the pathways patients take from first symptom to diagnosis. Such pathways have not been mapped out, so we sought to address this evidence gap.

## Methods

This study was nested within a retrospective case-control study aimed at identifying and quantifying clinical features of lung cancer [2]. All 247 primary lung cancer cases aged ≥ 40 years, living in Exeter Primary Care Trust, Devon, UK between 1998–2002, were identified from the cancer registry at the Royal Devon & Exeter Hospital and from computerised searches at all 21 general practices in Exeter. Anonymised copies of the GP's records, referral letters, specialist consultations and chest X-ray results were taken. Nine features of lung cancer were identified from the GP notes in the year before diagnosis: cough, haemoptysis, dyspnoea, fatigue, loss of weight, loss of appetite, clubbing, chest or rib pain and hoarseness. The pathway from the first consultation in primary care with a feature of cancer to diagnosis was mapped out. The date of diagnosis was taken as the date that histological proof was obtained, or in the few that were diagnosed without histology, the date that a respiratory physician gave the diagnosis [2]. As the intervals between first symptom and diagnosis were not normally distributed, medians and inter-quartile ranges were used for analysis, with median tests for significance testing.

## Results

**Figure 1:**
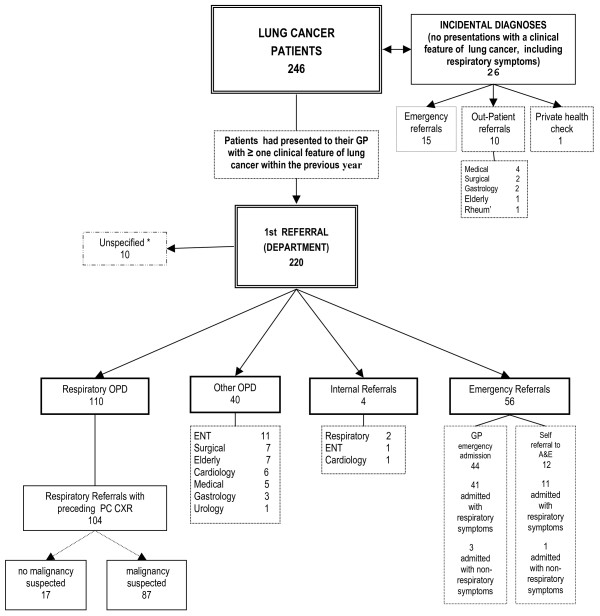
Lung cancer pathways from 1st consultation in primary care to diagnosis in secondary care.

A total of 247 patients were identified from the cancer registry and practice searches. One set of notes was unobtainable, so 246 were studied [2]. Of these, 171 (70%) were male, with a mean age of 72 years and 75 (31%) female, with a mean age of 68 years. The several possible pathways towards diagnosis are shown in Figure 1.

26 (11% of the total) of the patients had no feature of lung cancer recorded in their notes in the year before diagnosis: although some of them were referred to a specialist, lung cancer was not considered to be a likely explanation for their ill-health. Of the remaining 220 patients, 150 (61% of the total) were referred to an outpatient clinic, 110 (45% of the whole cohort, 73% of those referred to outpatients) of these to a respiratory physician. Four patients were referred by a hospital consultant to respiratory clinic (internal referrals). 56 patients (23% of the total) were admitted to hospital as an emergency, 52 of these with respiratory symptoms and four without respiratory symptoms. In 10 patients (4%) the precise referral pathway to secondary care was impossible to determine. The intervals between first symptom, referral and diagnosis are shown in Table 1.

**Table 1:** Intervals between symptom presentation, referral and diagnosis for lung cancer patients.

Department	Time in days between the two events (median, IQR)		
	
	First symptom presented to primary care to referral	Referral to diagnosis	First symptom presented to primary care to diagnosis
Outpatient referrals			
Respiratory (n = 110)	51 (17, 165)	29 (17, 61)	107 (55, 216)
Other (n = 40)	37 (13, 197)	66 (37, 110)	149 (73, 317)
Emergency inpatient admissions			
Self-referred (n = 11)	186 (38, 321)	15 (11, 37)	240 (48, 319)
GP referred (n = 45)	47 (6, 233)	16 (7, 59)	98 (45, 298)
Together (n = 56)	52 (7, 243)	16 (8, 40)	133 (45, 304)

The median (IQR) time to diagnosis for the 220 patients with a symptom of lung cancer was 121 (53, 261) days. For significance testing, the two types of emergency admissions – self-referral and GP referral – were merged. The interval from referral to diagnosis was highly significantly different across the three main routes, with emergency admissions having the shortest interval, and non-respiratory outpatient referrals having the longest: p < 0.001. Differences in the interval between first presentation to primary care with a symptom of lung cancer and referral were not significant across the three main referral routes; p = 0.96. In contrast, the difference in the intervals between first symptom presentation and diagnosis was highly significant; p < 0.001.

Of the 110 patients who were referred to the respiratory out-patients department 104 (95%) had had a chest X-ray taken in primary care. In 87 (84%) of these malignancy had been suggested in the X-ray report. Only 16 of 40 (40%) patients referred by the GP to other specialties had had a preceding X-ray, a significant difference; p < 0.001.

Of the 210 patients whose referral to secondary care is shown in Figure 1, 93 were before publication of the NICE guidance, and 117 afterwards. After publication, a higher proportion was referred to respiratory clinics, with 35 (37%) taking that route before guidance and 75 (64%) afterwards. Also after publication, the number of emergency admissions fell from 30 (32%) to 26 (22%), with smaller changes in the other categories. This change was significant: p = 0.003 (chi-squared with 4 degrees of freedom).

## Discussion

This study describes the pathways lung cancer patients took from first symptom recorded in primary care to diagnosis. There were three main routes. The majority (61%) were referred by their GP for specialist investigation as outpatients, though approximately one third of these were to non-respiratory departments. However, there were improvements in the proportion referred to respiratory clinics in later years. Overall this means that only 45% of the cohort (110 of 246) took the standard pathway. Almost all of these had had a chest X-ray taken in primary care. The next commonest route was by emergency admission, accounting for nearly a quarter of the cohort. The interval from the first recorded symptom and diagnosis was longest for misdirected referrals, and shortest for those admitted as an emergency. The difference in speed of diagnosis was in the interval after referral, not before referral. The final route was for those without a respiratory symptom, many of whom were also admitted as an emergency, but without predominant respiratory symptoms.

### Strengths and weaknesses

This is a single cohort from one area, and may not be typical. It is relatively small, though it did identify all the cancers occurring over a five year period. It overlaps the introduction of the first referral guidance for suspected cancer sent to GPs in 2000, and does not take account of the minor changes when these were revised in 2005 [8]. We do not know if the higher proportion referred to a respiratory clinic was due to the NICE guidance. However, it seems unlikely, given the unchanged ease of access to primary care chest X-rays, and that respiratory departments had been offering urgent appointments for suspected lung cancer (largely based on abnormal chest X-rays) for many years before the referral guidance was issued. We did not have mortality data, so could not assess if there was any association between the routes of diagnosis and clinical outcomes. Furthermore, the data originate from the GP records, and any omissions in medical recording of symptom, investigations or referrals will have weakened this study.

### Comparison with previous literature

No primary care study has identified what proportions of patients take the different routes to diagnosis. Much recent work has concentrated on outpatient referrals, and whether patients take the urgent or non-urgent route [9-11]. In a large UK study of cancers in 1999 and 2000, Allgar reported that 80% of lung cancer patients had seen their GP before diagnosis, a lower percentage than reported here [11]. These were self-reports rather than our GP reports, perhaps explaining part of the differences. That study also reported that patients who had seen their GP before diagnosis experienced longer delays in diagnosis than those who had not: this was again not seen in the study reported here. A Swedish cohort of 364 patients collected in secondary care found 7% to be asymptomatic, though how these patients had their cancer actually identified was not described [12]. Only one patient in the study reported here was symptomless; this is not surprising as few screening chest X-rays are now performed. Abnormal X-rays are the main trigger for referral to a respiratory physician, both in this study and another comparing urgent with non-urgent referrals [13].

### Implications of the findings

The main finding is that only a minority of patients take the standard route of GP referral to a respiratory physician. This is a smaller percentage of patients taking the standard route than for colorectal, breast or prostate cancers [14-16]. In part this reflects the group who were admitted as an emergency (though roughly the same proportion of patients with colorectal cancer are first diagnosed as part of an emergency admission) [17]. The other likely explanation is that lung cancer patients may be more systemically unwell, with respiratory symptoms either minor, or even absent. This may also explain those patients who were referred to other departments. These were also less likely to have been X-rayed. These patients all had a symptom associated with lung cancer, but other features may have predominated, making the GP omit an X-ray. Whatever the explanation, this finding is important, as most diagnostic initiatives have been concentrated upon the standard pathway.

Nearly a quarter of patients were admitted to hospital as emergencies. Emergency admissions have not previously been reported as a common route to diagnosis in lung cancer. Many of these admissions will have been for a respiratory infection, and the underlying cancer will only have become apparent because of delayed recovery, or an abnormality on the chest X-ray.

The third group was those without a respiratory symptom. It is arguable that this group of unexpected diagnoses is even smaller than reported, as many of these patients were ill enough to be admitted to hospital, even if lung cancer was not being considered. Every condition has atypical presentations; that these are relatively rare in lung cancer is helpful.

### Lessons for expediting diagnosis of lung cancer

Several messages arise from this study. The first is for GPs, in that some patients with lung cancer are referred to the wrong speciality, and their diagnosis is delayed as a result. This may be improved if there was a greater willingness to take a chest X-ray. The NICE guidelines encourage this practice, though it could be argued that the threshold for ordering an X-ray is set too high in those recommendations.

The median interval between first recording of the cancer symptom in the notes and eventual diagnosis is nearly four months; around half of this is delay is in the GP making a referral. This finding suggests that there is some potential for expediting diagnosis in symptomatic lung cancer patients. The median time from referral to diagnosis is short. This may reflect the smaller numbers of urgent referrals made to respiratory clinics when compared to the other common cancers. This is a major advantage of the chest X-ray. Those who have an abnormal X-ray – and thus qualify for urgent referral – have a much higher likelihood of cancer than their counterparts with possible breast or colorectal cancer. Thus, respiratory clinics should be able to offer an efficient service – this study suggests they do.

Thirdly, researchers who wish to expedite lung cancer diagnosis will have to consider the three different main routes to diagnosis. Concentrating on the standard pathway will miss around half of patients, including those with the greatest delays in diagnosis.

Finally, policymakers need to address how patients can be encouraged to attend when they have a lung cancer symptom [3,4]. Early presentation may obviate some of the emergency admissions, and allow more accurate direction of referrals. This is not an easy task, as many of the symptoms of cancer carry a low risk [2], and there is a fine dividing line between promoting early presentation, and a need to avoid overwhelming GPs and radiology departments.

## Competing interests

The authors declare that they have no competing interests.

## Authors' contributions

JB collected the data and performed the analyses.

WH supervised the study, and wrote the initial draft of the paper which both authors revised.

Both authors read and approved the final manuscript.

## Pre-publication history

The pre-publication history for this paper can be accessed here:


